# Artificial Neural Networks Fitting of Potential Energy Curves and Surfaces: The 1/R Conundrum

**DOI:** 10.1002/jcc.70220

**Published:** 2025-09-15

**Authors:** Siddhuram Rana, Uday Sankar Manoj, Upakarasamy Lourderaj, Narayanasami Sathyamurthy

**Affiliations:** ^1^ School of Chemical Sciences National Institute of Science Education and Research (NISER) Bhubaneswar, An OCC of Homi Bhabha National Institute Khurdha India; ^2^ Indian Institute of Science Education and Research Mohali Manauli India

## Abstract

Within the Born‐Oppenheimer approximation, the potential energy of a molecular system is written as a sum of electronic energy and nuclear‐nuclear repulsion energy terms. The potential energy surface (PES), computed ab initio, as a function of bond distances and bond angles, has traditionally been represented using analytic functions and/or interpolation methods. We show here that the ab initio computed *electronic* energy values of a molecular system can be fitted more accurately than the corresponding potential energy values using the artificial neural network methodology. The exact Coulombic internuclear repulsion energy can be added subsequently to the fitted electronic energy to obtain an accurate PES.

## Graphical abstract

ANN fit of electronic energies and adding internuclear repulsion gives accurate potential energy surfaces.

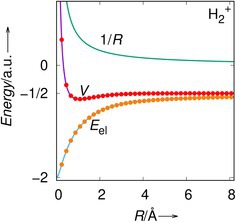

## 1 Introduction

Within the Born‐Oppenheimer approximation, the potential energy (V) for nuclear motion of a molecular system computed using ab initio electronic structure theoretical methods comprises the electronic energy ($E_{\text{el}}$) and the internuclear repulsion energy ($V_{\text{nu}}$) for a given geometry. For a diatomic molecule, for example, the electronic Schrödinger equation corresponding to state m is given by

(1) $$Ĥ\psi_{m}^{\text{el}}(r;R)=E_{m}^{\text{el}}(R)\psi_{m}^{\text{el}}(r;R)$$

where R is the internuclear distance, $E_{m}^{\text{el}}$ is the electronic energy, and $\psi_{m}^{\text{el}}(r;R)$ is the electronic wavefunction that depends on R parametrically. The potential energy ($V_{m}(R)$) for the nuclear motion in the $m^{\text{th}}$ electronic state of the diatomic molecule AB with nuclear charges $Z_{A}$ and $Z_{B}$ is then defined as

(2) $$\begin{array}{ll} V_{m}(R) & =E_{m}^{\text{el}}(R)+V_{\text{nu}}(R)\; \end{array}$$

(3) $$\begin{array}{ll} & =E_{m}^{\text{el}}(R)+\frac{Z_{A}Z_{B}}{R}\; \end{array}$$

in atomic units. Such a relationship is easily generalized to larger systems with R representing the collective internuclear coordinates and $V_{m}(\text{R})$ representing the potential energy surface (PES) for the molecular system in the $m^{\text{th}}$ electronic state.

A comparative review of 50 analytical representations of potential energy (PE) interaction of diatomic systems was published recently [1]. Those representations were designed largely to reconstruct the potential energy curves (PECs) from spectral data. Le Roy [2] had tried to automate the construction of the PECs. Underlying all those efforts is the realization that the short‐range interaction is repulsive and that the long‐range interaction is attractive. While such a representation for covalently bonded species is reflected in the Morse function [3] in the form of a difference between two exponentials, Lennard‐Jones potential [4] represents the van der Waals‐type interaction as the difference between two inverse powers of the internuclear separation R. Buckingham potential [5] combined the short‐range Pauli repulsion in the form of an exponential and the long‐range attraction in the form of inverse powers of R. Tang and Toennies [6, 7, 8] and others have tried to combine the Hartree‐Fock (exponential) potential for the short range and dispersion forces in the long range with a switching function connecting the two smoothly. Le Roy and collaborators have proposed modified Morse and modified Lennard‐Jones potentials to quantitatively account for the potential for covalently bonded species [9] and van der Waals type dimers [10].

Fitting an analytic function to a table of potential energy values computed ab initio and reported for different geometries of a polyatomic system continues to remain a challenge [11, 12, 13, 14]. Although several analytic functions have been proposed over the decades [15, 16, 17, 18, 19], several numerical methods [20] have been tried too, including the recent use of machine learning algorithms [21, 22, 23, 24, 25, 26, 27, 28, 29, 30, 31, 32, 33, 34, 35].

It is known that the electronic energy for a given state varies (smoothly) between the separated atom limit and the united atom limit of a diatomic species, for example. The (1/R) part of the potential energy, however, leads to a singularity as R goes to zero. As a result, fitting the potential energy curve becomes more difficult than necessary. It is worth mentioning here that in their landmark paper, Eyring and Polanyi [36, 37] made use of the electronic energy in constructing the potential energy surface for the H + H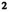 exchange reaction.

Engelke [38] had examined the efficiency of Coulomb‐subtracted (CS) analytic functions and concluded that the CS‐Dunham [39] expansion was highly accurate along the inner repulsive wall for a diatomic potential. CS‐Simon‐Parr‐Finlan (CS‐SPF) and CS‐Thakkar (CS‐T) expansions were no better than the original SPF [40] and Thakkar [41] expansions in representing the test diatomic potentials. CS‐Ogilvie‐Tipping (CS‐OT) and CS‐Pade approximants, on the other hand, were more accurate representations than the original functions [42, 43].

To extend the earlier Tang‐Toennies potential to the united atom limit, Warnecke et al. [44] found it necessary to define the electronic part of the potential by separating the Coulomb (1/R) part. Sheng et al. [45] extended their approach to represent the potential for all homonuclear and heteronuclear rare gas dimers.

In what follows, we demonstrate the use of the artificial neural network (ANN) method to fit the electronic energy values for diatomic and triatomic systems, and add the exact Coulombic nuclear‐nuclear repulsion to it subsequently to obtain more accurate potential energy values than the direct ANN interpolation of $V_{m}(\text{R})$ values.

## 2 Methodology

The basic methodology adopted in the ANN interpolation of potential energy values in one or more dimensions has been described elsewhere [46, 47, 48] and we refer to it as Scheme A. It involves choosing a set of neurons in one or more hidden layers and connecting them to the input geometries with different R values and the corresponding $V_{m}(\text{R})$ values for a training set (using a collection of adjustable weights and biases) that defines the network and gives the output $V_{\text{fit}}$. By comparing $V_{\text{fit}}$ with the known $V_{m}(\text{R})$ values and using a feedback algorithm and an iterative procedure, the input data is trained until a target mean‐squared‐error (MSE) is achieved. Then the predicted $V_{\text{fit}}$ for test data is compared with the corresponding $V_{m}(\text{R})$ values, and training is repeated with different networks until the desired quality of the fit is achieved. The ANN fits in the present study were performed using a modified sigmoid activation function and Bayesian regularization procedure employing the MATLAB program [49].

Scheme B proposed in this work involves four steps. (i) The $E_{m}^{\text{el}}(\text{R})$ values are obtained from the reported ab initio values of $V_{m}(\text{R})$ for a given geometry of the di‐/tri‐atomic system under consideration by subtracting the internuclear repulsion energy as follows:

(4) $$\begin{array}{ll} E_{m}^{\text{el}}(\text{R}) & =V_{m}(\text{R})-V_{\text{nu}}(\text{R})\; \end{array}$$

(5) $$\begin{array}{ll} & =V_{m}(\text{R})-\underset{j>i}{\sum }\underset{i}{\sum }\frac{Z_{i}Z_{j}}{R_{ij}}\; \end{array}$$

where $Z_{i}$ and $Z_{j}$ are the nuclear charges of the atoms i and j and $R_{ij}$ is the internuclear distance between the atoms i and j. (ii) As discussed in Scheme A, a major portion of the $E_{m}^{\text{el}}(\text{R})$ values and the corresponding **R** values is chosen as the training set, and the remaining forms the test data. The ANN model is defined by choosing a network consisting of a set of neurons in one or more hidden layers connected to the input R values and the corresponding $E_{m}^{\text{el}}(\text{R})$ values, which gives the output $E_{\text{fit}}^{\text{el}}$ for the training set using a collection of adjustable weights and biases. Training of the ANN model is done by comparing the computed $E_{\text{fit}}^{\text{el}}$ values with the known (ab initio) $E_{m}^{\text{el}}(\text{R})$ values and adjusting the weights and biases using a feedback algorithm until a target MSE is achieved. (iii) The quality of the resulting ANN fit is checked by predicting the $E_{\text{fit}}^{\text{el}}$ values for the test data set. The training is repeated with different choices of networks until the desired quality of the fit is achieved. (iv) The exact $V_{\text{nu}}(\text{R})$ values are added to the ANN fitted $E_{m}^{\text{el}}(\text{R})$ values to obtain the $V_{m}(\text{R})$ values for the desired geometry. As in Scheme A, the ANN fits were performed using a modified sigmoid activation function and Bayesian regularization procedure employing the MATLAB program [49].

It is important to verify that the potential energy functions generated using the electronic energy‐based fits (Scheme B) are sufficiently accurate for computing spectroscopic constants. To this end, we computed the vibrational bound states of the diatomic systems considered in this study (H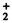 and H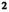) using the Fourier Grid Hamiltonian (FGH) method [50]. The ANN‐fitted potential energy functions were extended to the long‐range region by appending analytical interaction forms, smoothly connected via switching functions. For H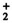, the long‐range form was taken from the work of Wind [51], and for H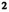, from Kolos and Wolniewicz [52]. Further details are provided in the Supporting Information.

## 3 Results and Discussion

### 3.1 H System

As discussed above, the electronic energy is a monotonically increasing function of the internuclear distance, R, for the H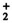 system in its ground electronic state. Following the work of Wind [51], we used 401 data points of $E^{\text{el}}$ values for different R values ranging from 0.026 Å to 10.583 Å to represent the electronic energy curve using different single‐ and double‐layered ANNs with 80% data used for training and 20% data for testing. In addition, the training data set for Scheme B included the electronic energy in the united atom limit (R=0). The ANN was trained with a target mean‐squared‐error (MSE) of 0.04 cm−2, for a maximum of 5000 epochs. The results of the various ANN fits (Scheme B) are listed in Table 1.

**TABLE 1:** Summary of the fits of the potential energy values (Scheme A) and electronic energy values (Scheme B) for H using different ANNs.

Network	RMSD (cm^−1^)		No. of epochs
	Training data	Test data	
Scheme A, Training: Test = 8:2			
10	18.30	19.00	5000
15	13.30	18.56	5000
20	31.13	27.68	5000
(5,5)	2.30	1.74	5000
(10,10)	1.14	1.14	5000
(20,20)	2.06	2.06	5000
Scheme B, Training: Test = 8:2			
10	0.20	0.19	1459
15	0.20	0.14	1503
20	0.20	0.22	2387
(5,5)	0.20	0.20	2071
(10,10)	0.20	0.17	282
(20,20)	0.20	0.17	793
Scheme B, Training: Test = 4:6			
10	0.01	0.23	5000
20	0.01	0.03	5000
30	0.01	0.04	5000

**FIGURE 1:**
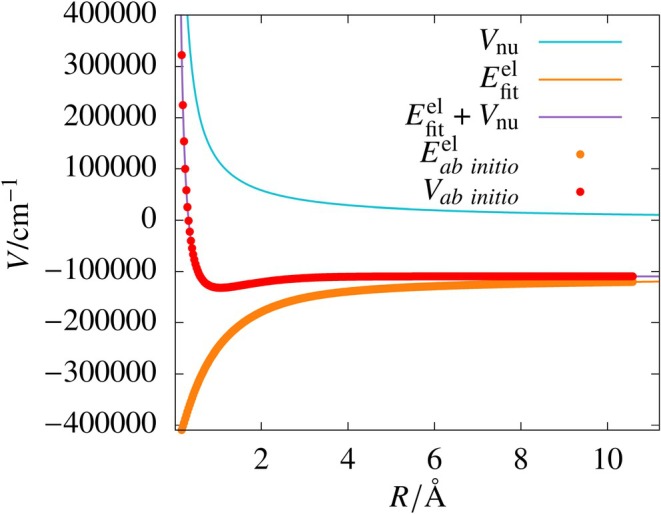
Plots of the *ab initio* potential ($V_{\text{ab initio}}$), the exact (1/R) term ($V_{\text{nu}}$), *ab initio* electronic energies ($E_{\text{ab initio}}^{\text{el}}$), and sum of ANN fit of electronic energies ($E_{\text{fit}}^{\text{el}}$) obtained from the single‐layered 15‐node network and $V_{\text{nu}}$ for H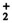 molecule.

Overall, the electronic energy fits are found to be of good quality. We can see that an increase in the number of nodes in the hidden layer increased the accuracy of the fit. Excellent accuracy was obtained for a single‐layer 15‐node network and a double‐layer (10, 10) network. The potential energy curve obtained using the electronic energy fit is shown in Figure 1. We can see that the fitted curve closely follows the raw data points without overfitting. For comparison, we fitted the potential energy data (Scheme A) using the same network configurations used for the electronic energy fits (Scheme B), and the results are given in Table 1. For all the networks, using Scheme A did not achieve the target MSE within 5000 epochs. The double‐layer networks performed better than the single‐layer networks with the best root‐mean‐squared deviation (RMSD) of 1.14 cm−1 as illustrated in Table 1. Further, electronic energy fits (Scheme B) were also performed for a smaller training data set with training: Test ratio of 4:6 using different single‐layer networks for the potential energy range from −130,000 to −93,000 cm−1. The fits were of good quality with a low RMSD value of 0.03 cm−1 for the test data when a 20‐neuron network was used.

To investigate if desirable accuracy could be achieved with fewer data points for the H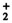 system, we carried out ANN fits using 101 and 201 data points. A training‐to‐test ratio of 8:2 was used, with training data points selected randomly. The results of the best fits are summarized in Table 2 and compared with those obtained using the complete data set of 401 points. The RMSD values for the test data set remained low for the Scheme B fits across all dataset sizes. For Scheme A, although the RMSD values for the training data set were smaller, they were larger for the test data set for the 201‐ and 101‐point sets. This was primarily due to large deviations in the repulsive region of the potential energy curve. To address this issue, we selected manually training sets with increased data density in the repulsive region, which led to significantly improved fits in Scheme A (Table S1). This result suggests that fitting schemes based on electronic energies may be better suited for regions of the potential energy curve with strong repulsion and large energy variations.

**TABLE 2:** Comparison of the best fits of the potential energy values (Scheme A) and electronic energy values (Scheme B) of H for three data sets of 401, 201, and 101 points^a^.

Selection of training data	random						manual	
Data points	401		201		101		201	101
Scheme	B	A	B	A	B	A	A	A
Network	15	(10, 10)	(5, 5)	(5, 5)	10	(5, 5)	(10, 10)	15
Training RMSD (cm−1)	0.20	1.14	0.20	0.40	0.20	1.56	0.20	0.81
Test RMSD (cm−1)	0.14	1.14	0.22	7972.74	19.66	20758.15	0.15	0.67

^a^ For Scheme A, 400, 200, and 100 points were used, since the R=0 data point was not included in the fits.

To demonstrate the utility of the proposed method of fitting electronic energies for computing accurate spectroscopic constants, we calculated the vibrational bound states of the H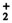 ground state using potential energy functions obtained from both Scheme A and Scheme B, with long‐range interactions added analytically (Figure S1). The results obtained using Schemes A and B were compared for datasets containing 401, 201, and 101 points. Tables 3 and S2 present the results of the bound state calculations. We can see that the number of bound states and their energies calculated through both schemes are in excellent agreement with each other.

**TABLE 3:** Comparison of bound states obtained using potential obtained from the ANN fits of the electronic energy with the (1/R) term added to it^a^ (Scheme B, $E_{n}^{B}$) with those obtained using the potential energy fit^b^ (Scheme A, $E_{n}^{A}$).

	H 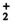			H 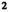		
n	$E_{n}^{B}$	$E_{n}^{A}$	$\Delta E=E_{n}^{B}-E_{n}^{A}$	$E_{n}^{B}$	$E_{n}^{A}$	$\Delta E=E_{n}^{B}-E_{n}^{A}$
0	−21375.92	−21375.32	−0.61	−36112.27	−36112.25	−0.02
1	−19183.98	−19183.92	−0.07	−31948.87	−31948.80	−0.07
2	−17119.07	−17119.53	0.46	−28020.77	−28020.76	−0.01
3	−15177.62	−15177.98	0.36	−24323.40	−24323.41	0.01
4	−13355.49	−13355.78	0.28	−20853.84	−20853.81	−0.02
5	−11649.87	−11650.12	0.24	−17611.05	−17611.02	−0.02
6	−10058.72	−10058.80	0.07	−14596.33	−14596.34	0.02
7	−8580.43	−8580.24	−0.18	−11813.72	−11813.75	0.03
8	−7213.82	−7213.48	−0.34	−9270.48	−9270.50	0.02
9	−5958.42	−5958.17	−0.25	−6977.81	−6977.86	0.05
10	−4814.62	−4814.65	0.02	−4952.15	−4952.21	0.07
11	−3783.70	−3783.98	0.27	−3216.59	−3216.59	0.00
12	−2867.79	−2868.08	0.30	−1802.96	−1803.01	0.04
13	−2069.83	−2069.87	0.04	−755.96	−755.99	0.03
14	−1393.69	−1393.42	−0.27	−137.57	−137.60	0.03
15	−844.40	−844.14	−0.26			
16	−428.46	−428.63	0.16			
17	−153.62	−153.72	0.10			
18	−23.37	−23.40	0.03			
19	−0.70	−0.69	−0.01			

^a^ The H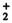 fit corresponds to a (10, 10) network using a dataset of 401 points, and the H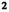 fit corresponds to a 10‐node single‐layer network using a dataset of 81 points.

^b^ The H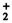 fit corresponds to a 15‐node single‐layer network using a dataset of 400 points, and the H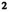 fit corresponds to a 25‐node single‐layer network using a dataset of 81 points.

### 3.2 H Ground State

As for the case of H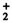, the electronic energy of H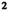 in its ground electronic state is also a monotonically increasing function of R and is simpler in features than the $V_{m}(R)$. Hence, it is expected that fitting the $E^{\text{el}}(R)$ values will be intrinsically simpler. We obtained the ground‐state potential energy data for H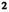 from the highly accurate work of Kolos and Wolniewicz [52]. The dataset consists of 81 potential energy points for R values ranging from 0.37 Å to 5.30 Å. 90% (72 points) of the data was used for training, and the remaining 10% (9 points) was used for testing the model. Different single‐ and double‐hidden‐layered networks were used for training. During training, the target MSE was set to 0.04 cm−2 for a maximum epoch of 10,000.

**FIGURE 2:**
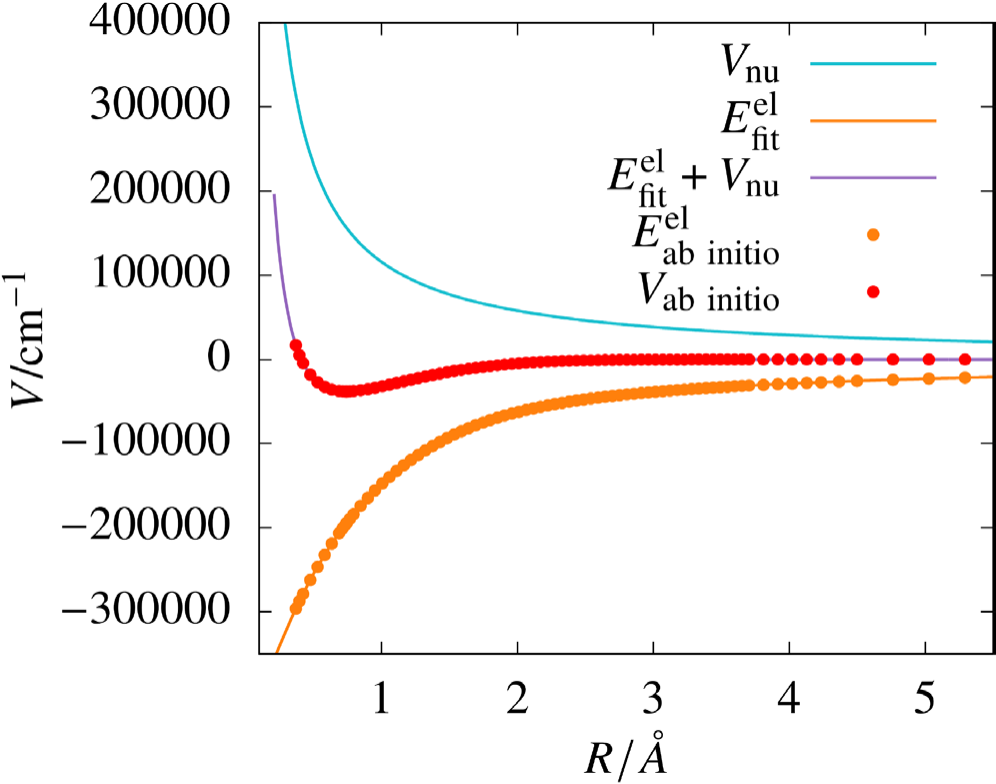
Plots of the *ab initio* potential ($V_{\text{ab initio}}$), the exact (1/R) term ($V_{\text{nu}}$), *ab initio* electronic energies ($E_{\text{ab initio}}^{\text{el}}$), and sum of ANN fit of electronic energies ($E_{\text{fit}}^{\text{el}}$) obtained from the single‐layered 10‐node network and $V_{\text{nu}}$ for H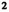 molecule.

**FIGURE 3:**
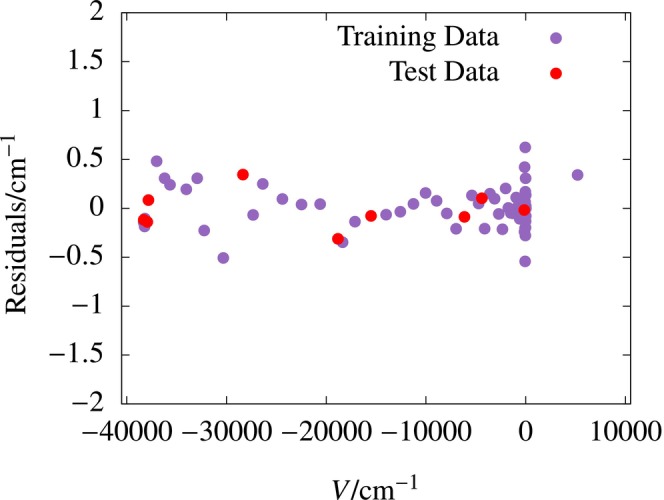
Residuals for the best single‐layer (25‐node) ANN fit using Scheme B for the H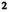 system.

We could see that for the H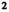 system, excellent fits to electronic energy values were obtained with small single‐layer networks. The best fit was obtained using a single‐layer 25‐node network having an RMSD of 0.18 cm−1 for the test data. The potential energy curve obtained using the electronic energy fit is plotted in Figure 2. The curve is smooth and it closely follows the ab initio data over the entire range of R. The residuals obtained for the single‐layer 25‐node ANN fit using Scheme B are shown in Figure 3.

To further illustrate the ease of fitting electronic energy values, the ab initio potential energy values of H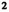 were fitted directly (Scheme A) using different ANNs, and the results are summarized in Table 4. Clearly, the ANN fits of electronic energy values (Scheme B) perform better than those of the potential energy values (Scheme A). The single‐layer 10‐node network gave the least test RMSD in Scheme A. In contrast, in Scheme B, all the single‐layer network models reported here gave lower test RMSDs compared to those for Scheme A.

**TABLE 4:** Summary of the fits of the potential energy values (Scheme A) and electronic energy values (Scheme B) of H using different ANNs.

Network	RMSD (cm^−1^)		No. of epochs
	Training data	Test data	
Scheme A, Training: Test = 9:1			
10	0.20	0.37	3814
15	0.20	0.54	1094
20	0.20	0.76	973
25	0.21	0.39	10,000
(5, 5)	0.20	0.40	9153
(10, 10)	0.20	0.56	586
(15, 15)	0.20	1.45	243
Scheme B, Training: Test = 9:1			
10	0.20	0.28	1611
15	0.20	0.25	208
20	0.20	0.25	81
25	0.20	0.18	472
(5, 5)	0.20	0.24	2252
(10, 10)	0.20	0.29	6148
(15, 15)	0.20	0.67	343

As in the case of H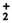, we calculated the bound states for the H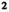 ground state using the potential functions obtained from Scheme A and Scheme B, with long‐range interactions included analytically (Figure S2). The results obtained using Schemes A and B were compared with each other. Tables 3 and S1 give the results of the bound state calculations. We can see that the number of bound states and their energies calculated by both schemes are in excellent agreement with each other.

### 3.3 H Excited State

Having demonstrated the utility of the ANN fitting of the ab initio electronic energy values for the ground electronic state of H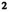, we explored its utility for the first excited state ($b^{3}\Sigma_{u}^{+}$), for which the potential and electronic energies are monotonic functions of R. We used a data set of 39 ab initio points reported by Jamieson et al. [53]. The data set covered R values ranging from 1 $a_{0}$ (0.529 Å) to 20 $a_{0}$ (10.583 Å). The data set was split into training and test sets in an 8:2 ratio, and the training was carried out for a maximum of 5000 epochs, with a target MSE of 0.04 cm−2. The results are summarized in Table 5.

**TABLE 5:** Summary of the fits of the potential energy values (Scheme A) and electronic energy values (Scheme B) for the $b^{3}\Sigma_{u}^{+}$ state of H using different ANNs.

Network	RMSD (cm^−1^)		No. of epochs
	Training data	Test data	
Scheme A, Training: Test = 8:2			
8	0.2	0.18	197
10	0.2	0.61	137
20	0.2	0.11	401
30	0.2	2.29	360
(4, 4)	0.2	0.2	140
Scheme B, Training: Test = 8:2			
8	0.2	0.22	1147
10	0.2	0.31	384
20	0.2	0.43	130
30	0.2	0.18	601
(4, 4)	0.2	0.26	222

**FIGURE 4:**
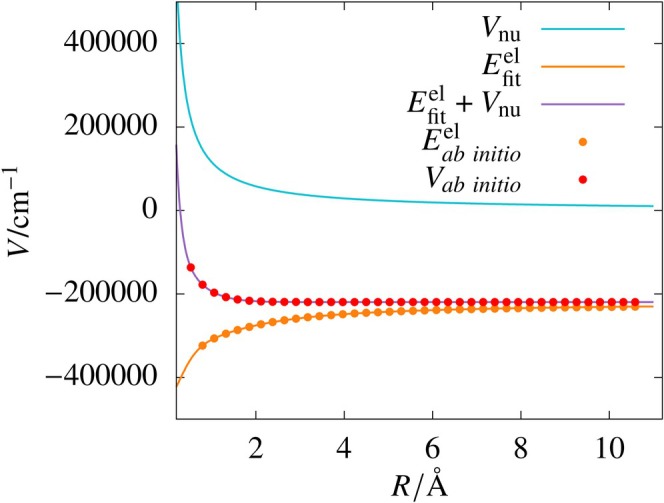
Plots of the *ab initio* potential ($V_{\text{ab initio}}$), the exact (1/R) term ($V_{\text{nu}}$), *ab initio* electronic energies ($E_{\text{ab initio}}^{\text{el}}$), and sum of ANN fit of electronic energies ($E_{\text{fit}}^{\text{el}}$) obtained from the (4,4) network and $V_{\text{nu}}$ for H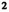 molecule in its first excited state.

Since V and $E_{\text{el}}$ are simple functions of R, we tried fitting with smaller network sizes, using one or two hidden layers. From the table, it can be seen that in Scheme A, all networks converge to the target MSE within 5000 epochs, with most achieving excellent RMSD values for the training and test sets. In Scheme B also, the target MSE is achieved within the maximum epochs for all the training cases, with RMSD values for the test set comparable to those in Scheme A. Figure 4 shows potential energy curve ($V_{\text{fit}}$) obtained using the best fit (network (4,4)) of electronic energies. The electronic energies ($E^{\text{el}}$) and nuclear repulsion term ($V_{\text{nu}}$) are also shown for completeness.

### 3.4 (HeH, He) System

**FIGURE 5:**
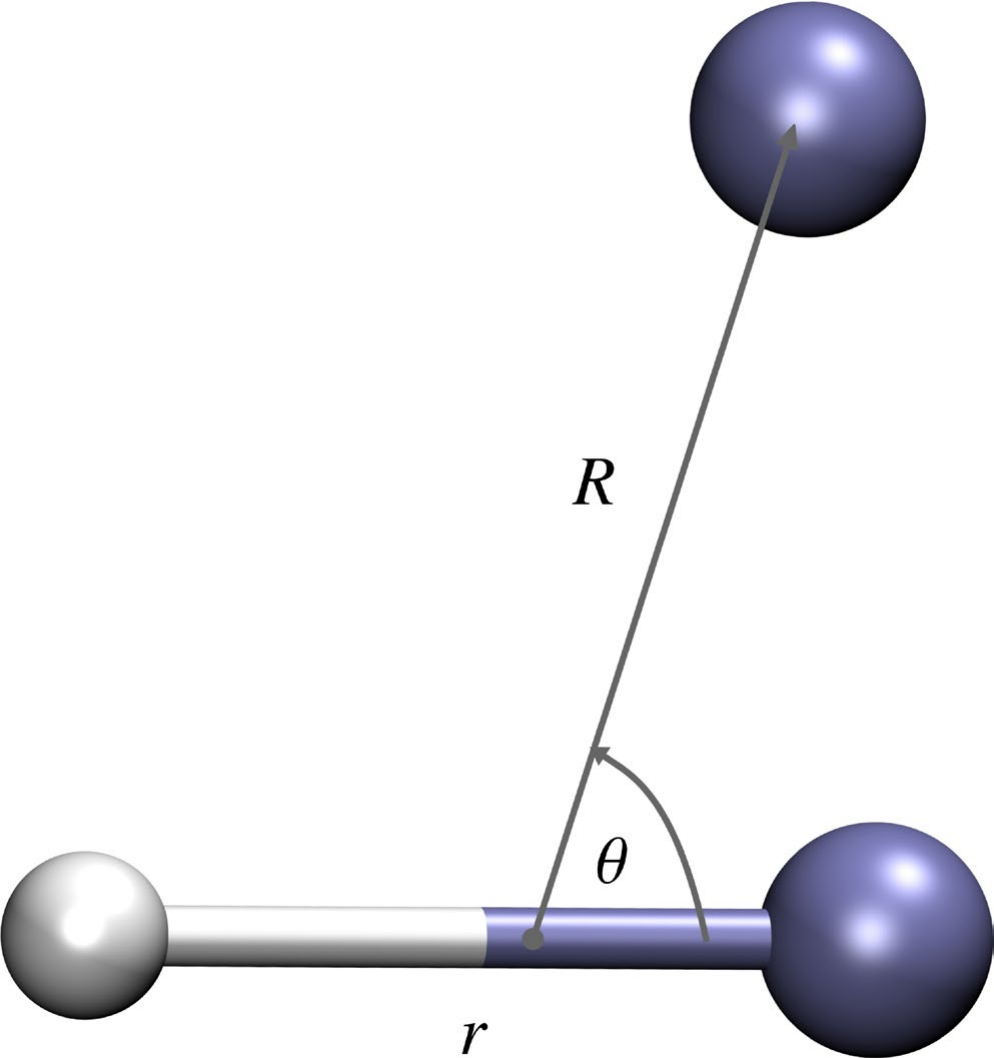
The definition of the Jacobi coordinates for the (HeH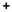, He) system. The violet spheres represent the He atoms, while the gray sphere represents the H atom.

Next, we considered a three‐dimensional (HeH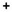, He) system to investigate if the fitting of electronic energy values (Scheme B) provides a more accurate representation of the PES than the direct fitting of V(R) values (Scheme A). The Jacobi coordinates, R, r, θ, used in the fitting of the PES are illustrated in Figure 5, where r is the He‐H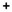 bond distance, R is the distance between the center of mass (X) of HeH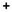 and He, and θ is the [He‐X‐He]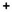 angle.

For this system, we used 15,049 electronic energy data points, where r ranged from 0.55 Å to 1.3 Å, R from 1.0 Å to 10.0 Å, and θ from 0° to 180°. The electronic energies were obtained from ab initio calculations at the CCSD(T)/CBS level of theory. This data set was divided into training (Set 1) and test data sets in the ratio 8:2, and the training was done for a maximum of 5000 epochs with a target MSE of 1 cm−2. A series of double‐hidden‐layer networks were used to fit the electronic energy values (Scheme B), and the results of the fits are reported in Table 6.

**TABLE 6:** Summary of the fits of the potential energy values (Scheme A) and electronic energy values (Scheme B) for the (HeH, He) system using different ANNs.

Network	RMSD (cm^−1^)		No. of epochs
	Training data	Test data	
Scheme A, Set 1			
(15, 15)	2.78	90.76	5000
(20, 20)	1.00	81.04	2125
(25, 25)	1.00	61.91	734
(30, 30)	1.00	82.73	1313
(40, 40)	1.00	92.88	1429
Scheme B, Set 1			
(15, 15)	4.30	24.02	5000
(20, 20)	2.66	10.63	5000
(25, 25)	1.00	7.83	1544
(30, 30)	1.00	11.98	2478
(40, 40)	1.00	10.58	3404
Scheme B, Set 2			
(15, 15)	4.11	10.87	5000
(20, 20)	1.00	1.74	2101
(25, 25)	1.00	1.21	707
(30, 30)	1.00	3.48	649
(40, 40)	1.00	6.21	477

We could see that the double‐layer network with 15 nodes each had a low RMSD of 4.3 cm−1 for the training set, but was unable to achieve the target MSE. After 5000 epochs, the RMSD value for the test data set stood at 24.02 cm−1. Increasing the nodes to 20 in each layer improved the RMSD value for both training and test data sets. However, it could not achieve the target MSE within 5000 epochs. The (25, 25) network in Scheme B gave an excellent fit with an RMSD of 7.83 cm−1 for the test data set in 1544 epochs. A further increase in the network size to 30 and 40 nodes in each layer achieved the target MSE within 5000 epochs with RMSD values of 11.98 and 10.58 cm−1, respectively, for the test data.

To compare the electronic energy fits to that of the potential energy fits, we trained the potential energy data set with different ANNs (Scheme A) using the same training: Test data ratio of 8:2 and the same networks as in Scheme B for a maximum of 5000 epochs. The RMSD values obtained from the different fits are given in Table 6. We can see that after 5000 epochs, the RMSD value for the training data set for the (15, 15) network was 2.78 cm−1, while that for the test data was 90.76 cm−1. Increasing the network size resulted in the training set reaching the target MSE of 1.0 cm−2 within 5000 epochs. However, they all had large RMSD values for the test data, indicating overfitting. Therefore, it is clear that for the (HeH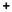, He) system, fitting the PES using electronic energy values (Scheme B) proves to be more efficient than fitting the potential energy data directly (Scheme A).

**FIGURE 6:**
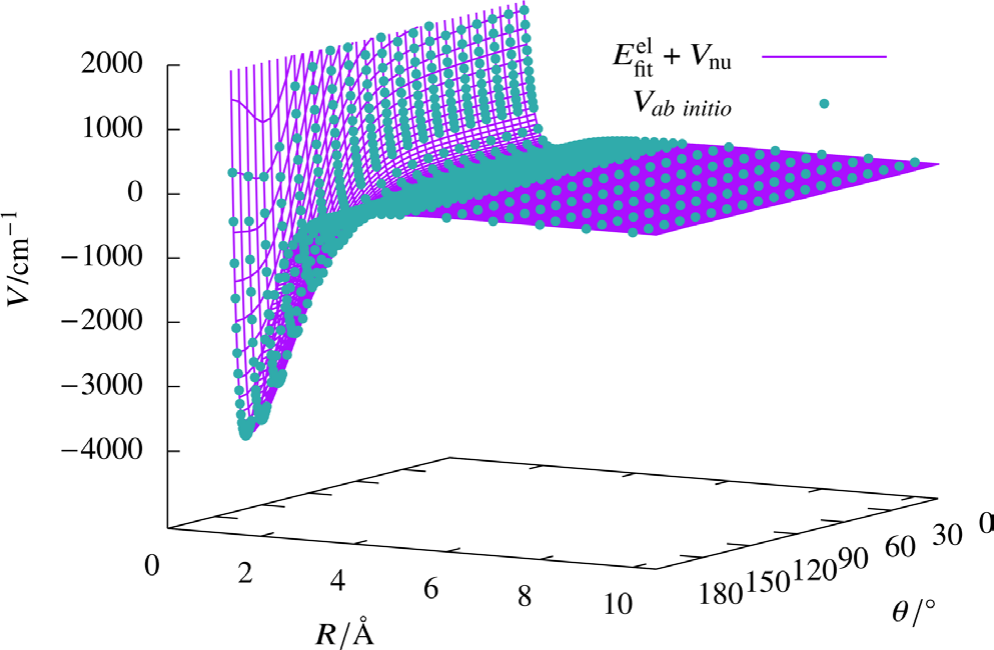
Potential energy surface plotted as a function of R and θ for the 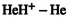 system with r = 0.774 Å obtained from *ab initio* data and from the sum of $V_{\text{nu}}$ with electronic energy values ($E_{\text{fit}}^{\text{el}}$) obtained from (25, 25) ANN fit (Scheme B).

Although the electronic energy fits (Scheme B) with the (25, 25), (30, 30), (40, 40) networks achieved the target MSE, the RMSD values for the test data were orders of magnitude higher compared to the RMSD of 1 cm−1 for the training set, indicating over‐fitting. Careful analysis of the residuals revealed that the residuals contributing to the larger RMSD values for the test data were due to extrapolation at the edge points. Hence, to improve the fit, selected points were added to make a new data set (Set 2), and ANN fits were performed on this set using different networks. We can see from Table 6 that the RMSD values for the test data set have significantly improved with the (25, 25) network yielding the best RMSD value of 1.21 cm−1 comparable to that for the training set. Thus, the best fit among the trials considered was the one corresponding to the (25, 25) network corresponding to Set 2. The quality and smoothness of the (25, 25) ANN fitted PES is illustrated in Figure 6 where the potential energy surface obtained by fitting the electronic energy values is compared with the input ab initio $V_{m}(\text{R})$ values. To check the local smoothness of the fit near the stationary points, we computed the global minimum energy point and its corresponding harmonic frequencies using the (25, 25) network ANN fit (Set 2). The minimum energy point had a linear structure as expected, and the geometrical parameters of R=1.66 Å, r=0.92 Å, and $\theta =180^{\circ }$ were found to be in close agreement with our raw data.

### 3.5 (He, H) System

**FIGURE 7:**
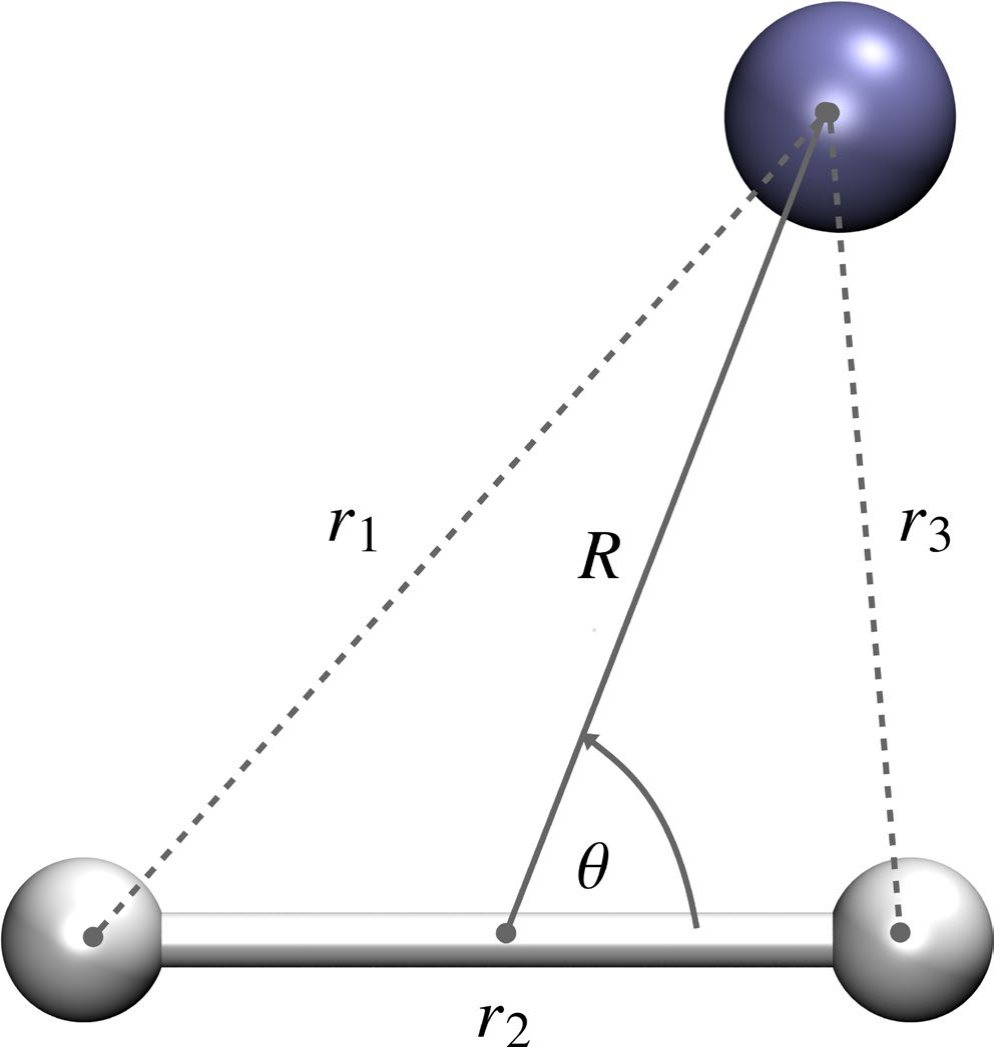
The definition of the coordinates used for the (He, H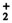) system. The violet sphere represents the He atom, while the gray spheres represent the H atom.

We then considered a reactive system to examine if the fitting of electronic energy values (Scheme B) can provide an accurate representation of the PES. The coordinate system used in the fit is shown in Figure 7.

**FIGURE 8:**
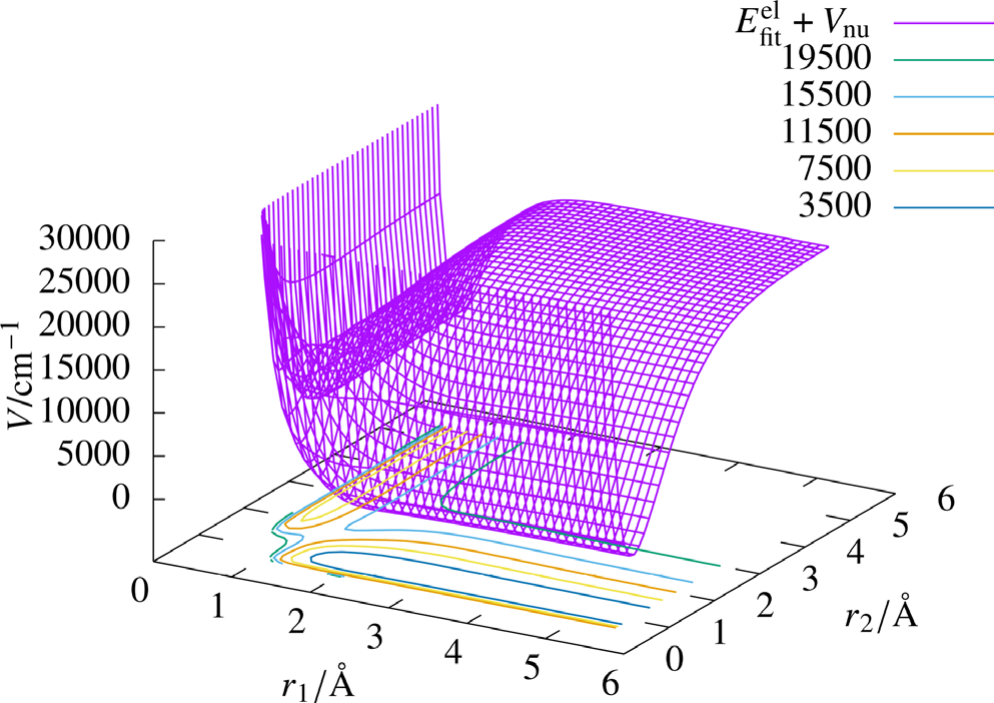
Potential energy surface for the He‐H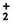 reactive system obtained from the (30, 30, 30) ANN fit using electronic energy values ($E_{\text{fit}}^{\text{el}}$) and $V_{\text{nu}}$ (Scheme B). The surface is plotted as function $r_{1}$ and $r_{2}$ with the angle between $r_{1}$ and $r_{2}$ fixed at $60^{\circ }$. Included in the figure are potential energy contours for the system.

We used 12,900 electronic energy data points, where $r_{1}$ and $r_{3}$ ranged from 0.5 $a_{0}$ to 10.0 $a_{0}$, and $r_{2}$ from 0.4 $a_{0}$ to 10.0 $a_{0}$. The electronic energies were obtained by subtracting the internuclear repulsion energy from the potential energy values obtained from the reported PES of Palmieri et al. [54]. The data was divided into training and test data sets in the ratio 8:2, and the training was done for a maximum of 5000 epochs with a target MSE of 0.04 cm−2. Different networks were tried to fit the electronic energy values (Scheme B), and the results of the fits are reported in Table 7. We can see that the best fit is obtained for the (30, 30, 30) network with an RMSD value of 0.79 cm−1 for the test data set, illustrating that the method of fitting electronic energy values can be extended to reactive systems also. The PES obtained from the (30, 30, 30) ANN fit is illustrated in Figure 8. We can see that the PES varies smoothly without any undulations.

**TABLE 7:** Summary of the fits of the electronic energy values (Scheme B) for the (He, H) system using different ANNs.

Network	RMSD (cm^−1^)		No. of epochs
	Training data	Test data	
Scheme B			
(30, 30)	1.85	3.92	5000
(40, 40)	0.37	2.36	5000
(50, 50)	0.21	1.91	5000
(20, 20, 20)	0.72	2.13	5000
(30, 30, 30)	0.21	0.79	5000

### 3.6 Angular Variations

It is worth investigating the quality of fitting using the proposed method for fitting electronic energies as a function of angular variables. To this end, we illustrate the utility of the method considering two cases: (i) Jacobi angle θ in HeH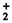 and (ii) torsional angle ϕ in ethane.

*i. Variation of* θ *in* (He,H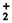)

**FIGURE 9:**
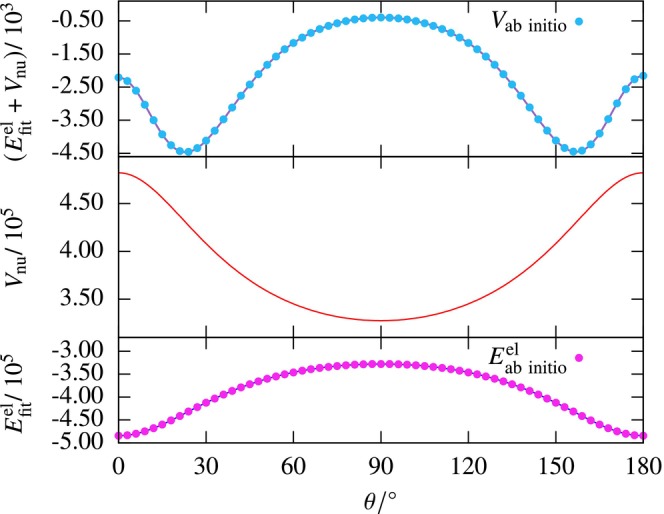
The variation of the fitted electronic energy (Scheme B), nuclear‐nuclear repulsion energy, and their sum as a function of θ for the (He, H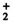) system. The energies are in cm−1 units.

Sixty‐one data points were generated for different values of θ (Figure 7) ranging from $0^{\circ }$ to $180^{\circ }$, with an increment of $3^{\circ }$, at fixed internuclear distances of $r_{2}=2.0\;a_{0}$ and $R=3.5\;a_{0}$, using the Palmieri potential energy surface [54]. The resulting dataset was then split into training and test sets in an 80:20 ratio and trained to an ANN model employing Schemes A and B. Table 8 summaries the results of the different fits obtained using Schemes A and B for the variation of energies with θ for (He, H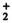) system. The best fit is obtained for the 15‐node single‐layered network. The variation of the fitted electronic energy (Scheme B, $E_{\text{fit}}^{\text{el}}$), nuclear‐nuclear repulsion energy, and their sum as a function of θ is shown in Figure 9. We can see that the $E_{\text{fit}}^{\text{el}}$ function varies smoothly from $\theta =0^{\circ }$ to $180^{\circ }$ reaching a maximum at $90^{\circ }$.

**TABLE 8:** Summary of the fits of the potential energy values (Scheme A) and electronic energy values (Scheme B) for the variation of θ in (He, H) system using different ANNs.

Network	RMSD (cm^−1^)		No. of epochs
	Training data	Test data	
Scheme A, Training: Test = 8:2			
10	0.20	0.24	176
15	0.19	0.54	34
20	0.11	0.33	30
25	0.20	0.26	107
30	0.12	2.27	11
35	0.19	0.81	27
40	0.18	1.54	10
(5, 5)	0.20	0.28	258
Scheme B, Training: Test = 8:2			
10	0.20	1.54	722
15	0.20	0.30	1193
20	0.20	0.45	64
25	0.20	1.85	186
30	0.20	10.31	453
35	0.20	24.39	120
40	0.20	0.70	379
(5, 5)	0.20	0.33	5747

*ii. Variation of Torsional Angle* ϕ *in Ethane*

**FIGURE 10:**
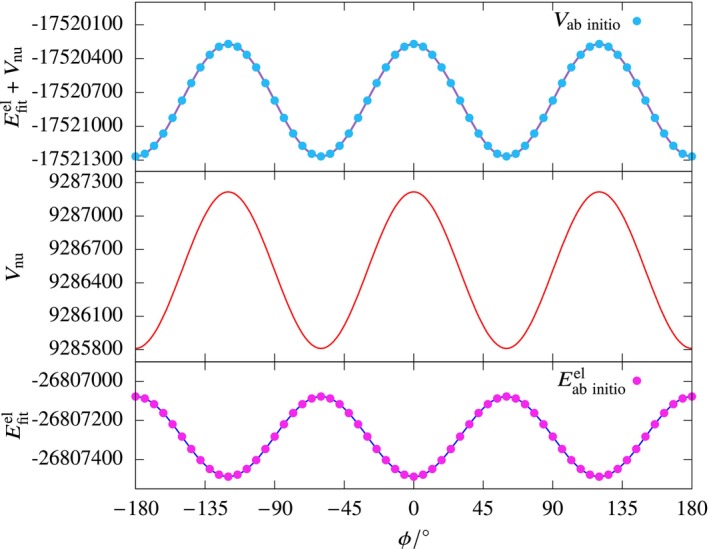
The variation of the fitted electronic energy (Scheme B), nuclear‐nuclear repulsion energy, and their sum as a function of *ϕ* for the ethane system. The energies are in cm^−1^units.

The potential energy curve as a function of the H–C–C–H torsional angle (ϕ) in ethane was generated using a rigid scan at the *ω*B97X‐D/def2‐TZVP level of theory. This involved rotating one of the methyl groups about the C–C bond. The torsional angle was varied from −180° to +180°, yielding 61 data points. The use of the *ω*B97X‐D functional is particularly suitable for ethane, as it accurately captures the subtle energy differences between staggered and eclipsed conformers due to its inclusion of long‐range dispersion corrections and correct asymptotic behavior. As discussed above, ANN fits were obtained for various networks following Schemes A and B with a training to test ratio of 8:2. The results are summarized in Table 9. We can see that for both Schemes A and B, a single‐layered network with more than 5 nodes is sufficient to produce accurate fits. A 35‐node single‐layered network gave the best results for Scheme A, while for the electronic energy fit (Scheme B), the 30‐node single‐layered network was the best fit. The plot of the fitted electronic energy (Figure 10) as a function of ϕ shows a variation of about 500 cm−1 in energy and is smooth. The large variation in the potential energy is dominated by the nuclear‐nuclear repulsion energy.

**TABLE 9:** Summary of the fits of the potential energy values (Scheme A) and electronic energy values (Scheme B) as a function of the torsional angle for ethane.

Network	RMSD (cm^−1^)		No. of epochs
	Training data	Test data	
Scheme A, Training: Test = 8:2			
10	0.20	1.03	47
15	0.19	1.40	44
20	0.20	1.36	42
25	0.17	1.49	17
30	0.14	1.14	7
35	0.06	0.61	15
40	0.19	0.89	46
Scheme B, Training: Test = 8:2			
10	0.20	0.63	109
15	0.20	0.89	16
20	0.19	0.74	31
25	0.18	0.67	7
30	0.05	0.23	6
35	0.16	0.61	5
40	0.19	0.88	72

## 4 Summary and Conclusion

It is shown that ANN fitting of electronic energies of molecular systems, followed by the addition of the applicable exact Coulombic repulsion energy to obtain the potential energy values (Scheme B), leads to more accurate results than directly fitting the computed ab initio potential energy values (Scheme A) for di‐ and triatomic systems investigated. Since the ANN fitting is, in principle, not system‐ and dimension‐dependent, it can be assumed safely that the adopted strategy would work for larger molecular systems too.

Here, we clarify that the reported ANN fit represents only the ab initio electronic energy values within the range considered and does not provide an asymptotically correct long‐range potential for any system discussed in this paper. Accurate representation of long‐range interactions on PESs is crucial for modeling processes such as scattering and ion–molecule reactions. Traditional approaches typically rely on physically motivated analytic forms, including multipole expansions and dispersion terms [11, 55, 56]. While machine learning methods can achieve high accuracy for interpolation, they often fail to capture correct asymptotic behavior unless explicitly constrained. One solution to this problem, in the context of the fitting electronic energies proposed here, is range separation, where machine learning models describe short‐range interactions, and long‐range terms are included analytically [57, 58, 59, 60]. Other physics‐based machine learning schemes have been proposed to describe the long‐range interactions accurately [61, 62, 63, 64].

Additionally, one may be concerned about the accuracy and the reliability of ANN‐fitted electronic energy and hence the potential energy values for determining stationary points on the potential energy surface and for yielding dynamical results. Several studies have been carried out over the years that demonstrate the utility of ANN‐fitted PESs for several systems. See, for example, Biswas et al. [65, 66], Giri et al. [59]. Therefore, we are not repeating them here.

## Conflicts of Interest

The authors declare no conflicts of interest.

## Supporting information




**Data S1**: Supporting Information.

## Data Availability

The data that support the findings of this study are available from the corresponding author upon reasonable request.
