# Supplementary material for: Artificial Neural Networks Fitting of Potential Energy Curves and Surfaces: The 1/R Conundrum
Source: J Comput Chem. 2025 Sep 15;46(24):e70220. doi: 10.1002/jcc.70220 (PMC12434390; doi:10.1002/jcc.70220)
Supplement: Supplementary file 1 — Data S1: Supporting Information. [file JCC-46-0-s001.pdf]

Supplementary Material for:
Artificial Neural Networks Fitting of Potential Energy Curves and Surfaces: The 1/R Conundrum

Siddhuram Rana[1], Uday Sankar Manoj[1], Upakarasamy Lourderaj[1], Narayanasami Sathyamurthy[2]

[1]School of Chemical Sciences
National Institute of Science Education and Research (NISER) Bhubaneswar
An OCC of Homi Bhabha National Institute, P.O. Jatni, Khurdha 752050, Odisha, India,

[2]Indian Institute of Science Education and Research Mohali
SAS Nagar, Manauli 140306, Punjab, India

Correspondence: U Lourderaj (u.lourderaj@niser.ac.in), N Sathyamurthy (nsathyamurthy@gmail.com)

1 Long range interaction energy

1.1 H_2^+ system

Analytical form of the long-range potential (V_{LR}) for H_2^+ is given by:

$$V_{\mathrm{LR}}(R) = -\frac{1}{2} + \left(\frac{9}{4}\right) R^4 \tag{1}$$

where R is the internuclear distance in bohr.

A smooth switching function $S(R)$ is defined as:

$$S(R) = \frac{1}{1 + \exp\left(\frac{R-18.0}{0.5}\right)}. \tag{2}$$

The final potential $V_{\mathrm{final}}(R)$ is then constructed by appending the neural network-fitted potential $V_{\mathrm{ANN}}(R)$ with the long-range potential:

$$V_{\mathrm{final}}(R) = S(R) \cdot V_{\mathrm{ANN}}(R) + [1 - S(R)] \cdot V_{\mathrm{LR}}(R) \tag{3}$$

For the bound state calculations, the internuclear distance R was varied from $R_{\mathrm{min}} = 0.1\ a_0$ to $R_{\mathrm{max}} = 100\ a_0$. All bound state energies were obtained using the Fourier Grid Hamiltonian (FGH) method.

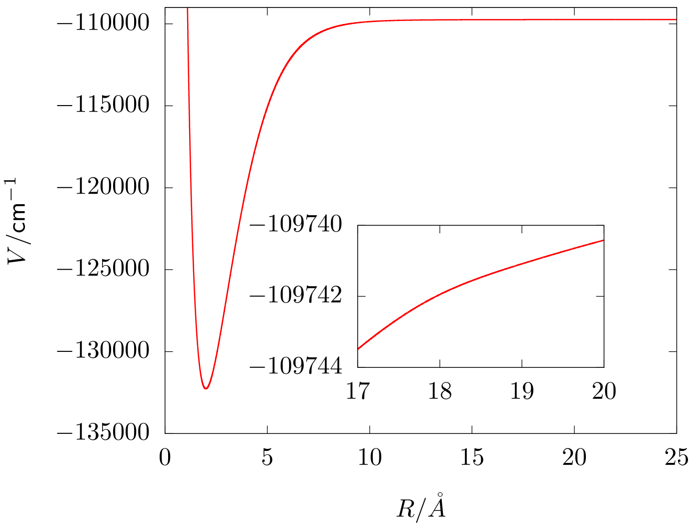

Fig. S1: Potential energy curve for H_2^+ system including long-range interaction.

1.2 H_2 system

The long-range potential V_{LR} for the hydrogen molecule is given by:

$$V_{\mathrm{LR}}(R) = -\left(\frac{C_0}{R^6} + \frac{C_1}{R^8} + \frac{C_2}{R^{10}}\right) \tag{4}$$

where the coefficients are: $C_0 = 6.499030$, $C_1 = 124.3990$, and $C_2 = 1135.210$ and R is the internuclear distance in bohr.

A smooth switching function $S(R)$ is defined as:

$$S(R) = \frac{1}{1+\exp\left(\frac{R-9.0}{0.2}\right)}. \tag{5}$$

The final potential $V_{\mathrm{final}}(R)$ is constructed as:

$$V_{\mathrm{final}}(R) = S(R) \cdot V_{\mathrm{ANN}}(R) + [1 - S(R)] \cdot V_{\mathrm{LR}}(R) \tag{6}$$

For bound state calculations, the internuclear distance R was varied from $R_{\mathrm{min}} = 0.56\ a_0$ to $R_{\mathrm{max}} = 28\ a_0$. All bound state energies were computed using the FGH method.

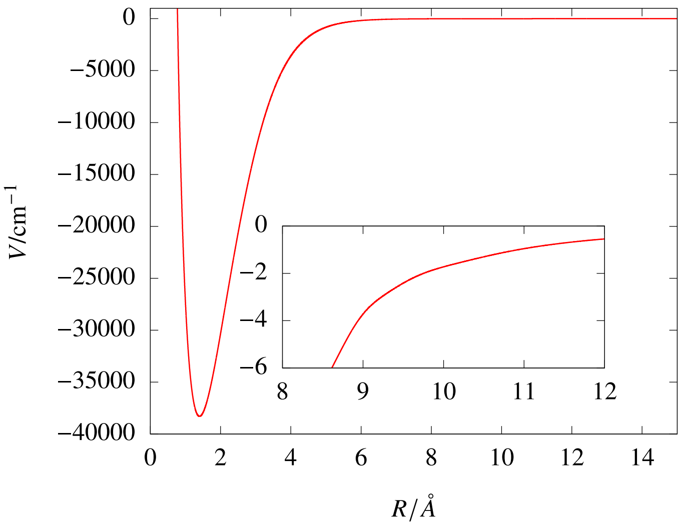

Fig. S2: Potential energy curve for H_2 system including long-range interaction.

2 ANN fits for data sets with 201 and 101 points

Table S1: Comparison of the ANN fits of the potential energy values (Scheme A[a]) and electronic energy values (Scheme B) of H_2^+ for two sets of data points: 201, and 101

	Scheme A: RMSD (cm^{-1})		Scheme B: RMSD (cm^{-1})	
Network	Training data	Test data	Training data	Test data
Number of data points = 201				
10	8.66	4.92	0.20	0.33
15	0.61	0.24	0.20	0.87
20	0.30	0.31	0.20	0.90
(5, 5)	1.21	0.80	0.20	0.22
(10, 10)	0.20	0.15	0.20	0.38
(20, 20)	3.23	10.53	0.20	0.56
Number of data points = 101				
10	5.77	7.16	0.20	0.18
15	0.81	0.67	0.25	0.33
20	11.13	36.70	0.20	0.25
(5, 5)	1.01	0.94	0.20	0.23
(10, 10)	0.51	5.12	0.20	0.59
(20, 20)	1.67	2.69	0.20	0.17

[a] For Scheme A, the data sets consisted of 200 points and 100 points, since the data point corresponding to $R = 0$ was not included in the fits.

3 Bound state calculations

Table S2: Comparison of bound states of H_2^+ obtained using potential obtained from the ANN fits of the electronic energy with the $(1/R)$ term added to it[a] (Scheme B, E_n^B) with those obtained using the potential energy fit[b] (Scheme A, E_n^A) for two data sets of points: 201 and 101

	Number of data points = 201			Number of data points = 101		
n	E_n^B	E_n^A	$\Delta E = E_n^B - E_n^A$	E_n^B	E_n^A	$\Delta E = E_n^B - E_n^A$
0	-21375.85	-21375.92	0.07	-21375.94	-21375.19	-0.75
1	-19183.82	-19183.80	-0.02	-19183.77	-19184.54	0.76
2	-17119.19	-17119.23	0.03	-17119.11	-17120.12	1.01
3	-15177.66	-15177.55	-0.11	-15177.56	-15178.19	0.63
4	-13355.60	-13355.42	-0.19	-13355.51	-13355.57	0.07
5	-11650.11	-11649.99	-0.12	-11650.01	-11649.62	-0.40
6	-10058.91	-10058.91	0.00	-10058.83	-10058.20	-0.62
7	-8580.39	-8580.47	0.08	-8580.34	-8579.76	-0.58
8	-7213.59	-7213.68	0.09	-7213.56	-7213.27	-0.30
9	-5958.20	-5958.25	0.05	-5958.19	-5958.31	0.12
10	-4814.58	-4814.59	0.01	-4814.57	-4815.11	0.54
11	-3783.86	-3783.82	-0.04	-3783.83	-3784.67	0.83
12	-2867.99	-2867.91	-0.08	-2867.92	-2868.80	0.88
13	-2069.88	-2069.81	-0.07	-2069.78	-2070.37	0.59
14	-1393.55	-1393.57	0.02	-1393.47	-1393.50	0.03
15	-844.27	-844.36	0.09	-844.29	-843.74	-0.55
16	-428.60	-428.58	-0.03	-428.64	-428.14	-0.51
17	-153.69	-153.62	-0.07	-153.59	-153.99	0.39
18	-23.38	-23.39	0.01	-23.41	-23.23	-0.18
19	-0.69	-0.69	0.00	-0.70	-0.76	0.06

[a] Best fits that correspond to (5,5) and 10 networks using dataset of 201 points and 101 points, respectively.
[b] Best fits that correspond to (5,5) and (5,5) networks using dataset of 200 points and 100 points, respectively, for Scheme A. The data point corresponding to $R = 0$ was not included in the fits.
